# Use of a Polylactic Acid Membrane for Wound Care After Severe Herpes Zoster (Shingles): A Case Report

**DOI:** 10.3390/reports9040362

**Published:** 2026-10-19

**Authors:** Víctor Manuel Loza-González, Patricia Aurea Cervantes-Báez, Andrea Paola Saucedo-Quintero, José Luis Ramírez-GarcíaLuna, Mario Aurelio Martínez-Jiménez

**Affiliations:** 1 Wound Clinic of the Regional High-Specialty Hospital “Ignacio Morones Prieto”, San Luis Potosí 78220, SLP, Mexico; vic.loza@hotmail.com (V.M.L.-G.); paty.cervantesb@hotmail.com (P.A.C.-B.); andreasaucedo1019@gmail.com (A.P.S.-Q.); 2 Division of Experimental Surgery, Faculty of Medicine, McGill University, Montreal, QC H3G 1A4, Canada; jose.ramirez-garcialuna@mail.mcgill.ca

**Keywords:** herpes zoster, shingles, polylactic-acid-based biomaterials, skin substitute, wound healing, case report

## Abstract

**Background and Clinical Significance:** Herpes zoster (HZ) results from reactivation of latent varicella-zoster virus and disproportionately affects older adults. Antiviral therapy and systemic analgesia are well established, but local wound care of the eroded skin remains largely empirical: no dressing has been validated in controlled trials for this indication, and denuded HZ lesions are frequently mismanaged with dry gauze that adheres to the wound bed and aggravates pain. **Case Presentation:** An 85-year-old man with no known chronic disease presented with a one-week history of thoracic HZ unresponsive to outpatient antiviral therapy, requiring admission for uncontrolled pain (9/10 on the Numeric Rating Scale, NRS), vomiting, asthenia, and hyporexia. Examination revealed confluent, superficial-partial-thickness erosions across the T6–T8 dermatomes (12.5 × 24 cm). After a negative wound culture, a single polylactic-acid-based membrane (Suprathel^®^250) was applied over the entire denuded area. Pain decreased from 9/10 to 2/10 on the NRS within seven days and complete re-epithelialization was observed by day 12, under concurrent antiviral and systemic analgesic therapy and the patient resumed independent daily activities by the third week without scarring. **Conclusions:** In this single uncontrolled case, pain reduction and wound closure followed one application of a polylactic acid membrane to an extensive HZ lesion. Because antiviral and analgesic therapy were given concurrently and HZ improves spontaneously over time, no causal or comparative inference is possible; the observation is hypothesis-generating and supports further controlled investigation. Suprathel^®^ is CE-marked and FDA-cleared for burn and wound care, so its use for HZ erosions extends an established burn indication.

## 1. Introduction and Clinical Significance

Herpes zoster (HZ), commonly known as shingles, results from reactivation of latent varicella-zoster virus (VZV, human herpesvirus 3), which persists in a non-replicating state within dorsal root and cranial nerve ganglia after primary chickenpox infection. Reactivation is thought to reflect a decline in VZV-specific cell-mediated immunity, whether due to physiological immunosenescence, psychological stress, or an immunosuppressive condition or treatment. Clinically, HZ evolves through a pre-eruptive (prodromal) phase of localized dysesthesia, an acute eruptive phase marked by a dermatomal maculopapular-vesicular rash, and a chronic phase in which pain may persist as post-herpetic neuralgia (PHN) [1,2].

The global burden of HZ is substantial and rising as populations age. A 2025 analysis of the Global Burden of Disease 2021 dataset covering 204 countries estimated that varicella and HZ cases increased by 19.0% between 1990 and 2021 (from 72.8 to 86.7 million), with age-standardized incidence remaining essentially stable while high-income regions saw a resurgence attributable to population aging [3]. Regional hospital-based series report similarly elevated figures: an Italian cohort of 47,933 HZ-related hospitalizations between 2011 and 2023 found an overall incidence of 6.46 per 1000 person–years, rising to 9.12 per 1000 person–years among adults over 65 [4]. Lifetime risk approaches one in three individuals, and may reach 50% among those who live into their ninth decade [2].

Several factors increase both the likelihood and severity of HZ. Advanced age is the single strongest predictor, reflecting progressive decline in VZV-specific immunity [2]. Diabetes mellitus is independently associated with a higher risk of HZ and of HZ-related hospitalization: a 10-year observational study from Piedmont, Italy, found that among diabetic patients, age over 65, obesity (BMI > 30), and poor glycemic control (HbA1c > 8.0%) increased the risk of HZ hospitalization, with diabetic patients facing a 40% higher rehospitalization risk and a 25% higher risk of severe complications compared with non-diabetic patients [5]. Other chronic conditions linked to reduced cell-mediated immunity malignancy—chronic kidney disease and autoimmune disease—have also been associated with more severe HZ courses and higher in-hospital mortality, particularly in patients over 80 years old [4]. Our patient, notably, had none of these comorbidities, which makes the severity of his presentation extensive multidermatomal involvement requiring hospital admission somewhat atypical and underscores that advanced age alone can be sufficient to produce a severe course.

Systemic antiviral therapy (acyclovir, valacyclovir, or famciclovir) remains the cornerstone of acute management and is most effective when started within 72 h of rash onset, although treatment may still offer benefit later if new vesicles continue to appear or if complications such as ophthalmic or neurological involvement are present [2]. Antivirals reduce the duration of viral shedding, accelerate lesion resolution, and lower the risk of PHN, but they do not directly address the cutaneous erosions that result once vesicles rupture.

By contrast, local wound care of the denuded skin in HZ has received comparatively little systematic attention. The eroded areas that follow vesicle rupture behave, in practice, as partial-thickness wounds: they are painful, prone to fluid loss, and vulnerable to secondary bacterial infection, which can itself precipitate rare but severe complications such as necrotizing fasciitis [6]. Historically, these lesions were treated with drying solutions and powders on the assumption that the rash is self-limited within two to three weeks; however, histopathological evidence that herpetic lesions extend into the dermis and behave as true partial-thickness wounds rather than superficial epidermal defects (demonstrated for herpes simplex lesions and extrapolated to HZ) has prompted growing interest in occlusive, moisture-retentive dressings as an alternative to traditional dry gauze [7]. A comprehensive literature review of patient-centered HZ wound outcomes found that healing time ranged from 2 to 7 weeks when lesions were dressed with a moisture-retentive dressing, compared with 9 to 10 weeks when lesions were left air-exposed [8]. Despite this, no dressing has been validated in a randomized controlled trial specifically for HZ, and a registered clinical trial (NCT04258930) comparing hydrocolloid occlusion, silicone gel, and standard drying-solution care for acute HZ remains, to our knowledge, the only registered controlled study addressing this question directly [9].

Suprathel^®^250 (ST250; PolyMedics Innovations GmbH, Denkendorf, Germany) is a thin, autoadhesive, acellular, biodegradable skin substitute composed of a synthetic polylactide-based copolymer (lactide 73–80%, trimethylene carbonate 12–16%, and caprolactone 8–11%) [10]. ST250 is a CE-marked medical device marketed in Germany and across the European Union for the treatment of partial-thickness burns and skin graft donor sites, that is, an established product in standard burn care rather than an experimental protocol, and it also holds U.S. FDA 510(k) clearance as a wound and burn dressing (K090160). It was developed for burns and is now used across a range of complex skin wounds [11]. In burn studies, ST250 causes significantly less pain during dressing changes than traditional paraffin-impregnated gauze, while producing comparable healing times and re-epithelialization rates [12,13,14]. Because it adheres directly to the wound bed and is gradually resorbed as the skin re-epithelializes underneath it, ST250 does not require the frequent dressing changes that make conventional gauze so poorly tolerated on denuded, hyperalgesic skin a property with direct relevance to the pain that dominates the clinical picture of acute HZ [12]. More recently, PLA-based matrices have also been applied outside the burn setting: a 2025 single-center European case series reported their use in chronic, therapy-resistant wounds of diverse etiology, describing favorable integration into the wound bed and a contribution to wound closure even in previously stalled lesions [15]. To our knowledge, however, no prior report has specifically examined the use of a polylactic acid membrane for the cutaneous lesions of HZ. We describe a case in which a single application of ST250 was associated with rapid pain relief and complete wound closure in a patient with extensive dermatomal HZ, and we place this outcome in the context of previously published wound-care strategies for HZ.

## 2. Case Presentation

### 2.1. Patient Information

An 85-year-old male, originally from San Luis Potosí and independent in his activities of daily living prior to this illness, presented to the emergency department with no history of diabetes mellitus, cardiovascular disease, malignancy, immunosuppressive therapy, or prior reported episodes of herpes zoster (HZ). He had no history of varicella-zoster virus vaccination and denied high-risk behaviors. He presented with a one-week history of a severe painful thoracic rash with edema and painful, crusted vesicular lesions affecting the left side across the mid-back along the T6–T8 dermatomes. Despite having been managed as an outpatient with oral antiviral therapy and topical care (Valacyclovir 500 g every 4 h and lidocaine 5% patch 1 patch every day) his condition progressively deteriorated, manifesting as severe neuropathic pain, persistent vomiting, severe asthenia, hyporexia, and inability to tolerate oral intake. He denied fever, chills, or shortness of breath. He lived with family members who assisted with his care during the acute presentation.

### 2.2. Clinical Findings

**Figure 1:**
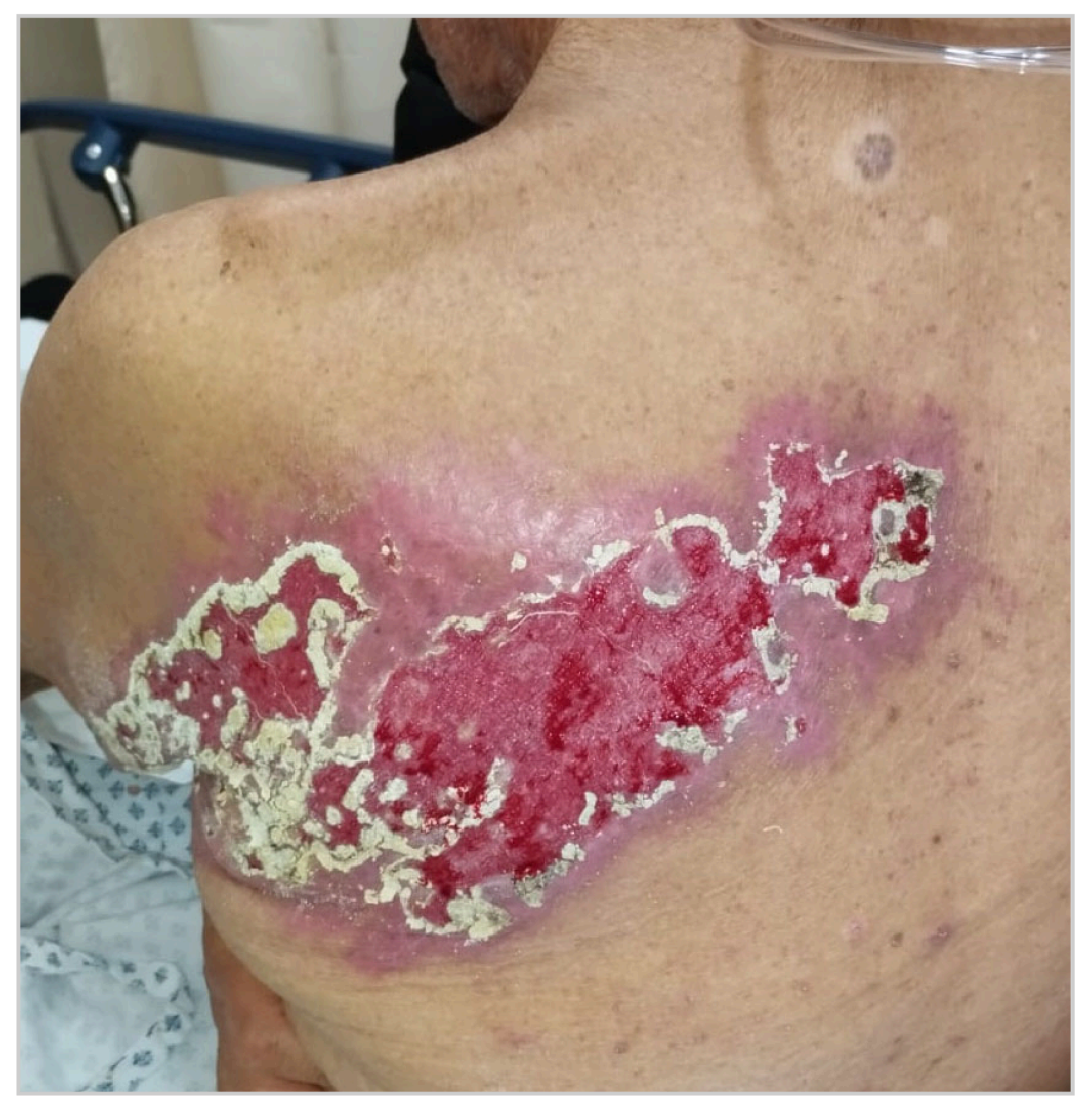
Extensive lesion with confluent areas of erosion across the T6–T8 dermatomes.

On admission, vital signs were within normal limits: body temperature was 36.7 °C, blood pressure was 126/88 mmHg, pulse rate was 76 beats per minute, oxygen saturation was 97%, and respiratory rate was 16 breaths per minute. He was alert, oriented, and conscious, but restless due to severe pain and burning at the site of the lesions. Systemic examination, including the respiratory and cardiovascular systems, showed no abnormalities. Dermatological examination revealed a large confluent area of erosion with a well demarcated dermatomal distribution across T6–T8 on the left hemithorax, extending from the paravertebral region anteriorly toward the midline (Figure 1). The lesion measured approximately 12.5 cm × 24 cm and presented with the clinical appearance of a superficial partial-thickness (second-degree) burn, erythematous wound bed with loss of epidermis and scattered residual vesicle roofs, but without evidence of full-thickness skin loss, active bleeding, or purulent discharge. The surrounding skin was edematous and tender to palpation. No satellite lesions, mucosal involvement, or signs suggestive of disseminated disease were observed. Pain was rated as 9/10 on the Numeric Rating Scale (NRS), described as a constant burning sensation with superimposed sharp exacerbations on contact with clothing or bedding, which significantly disrupted his sleep and rendered him unable to dress or bathe independently.

Components of the laboratory exam include complete blood count with differential, basic metabolic panel, and inflammatory markers showed no systemic abnormalities suggestive of bacterial infection or sepsis. White blood cell count was normal at 7620/mm^3^ with 60.8% neutrophils, 26.6% lymphocytes, and 12.5% monocytes, while C-reactive protein (CRP) was elevated at 40.3 mg/L (reference < 5 mg/L). Other routine biochemical parameters were within normal limits. Renal function was serum creatinine 1.1 mg/dL and estimated GFR 74 mL/min/1.73 m^2^, which was taken into account for valacyclovir dosing. The diagnosis of herpes zoster was established clinically based on history, characteristic dermatomal distribution, and lesion morphology. No virological confirmation (VZV PCR or direct fluorescent antibody testing) was obtained, as the unilateral dermatomal distribution and the morphology of the lesions were considered diagnostic, and no sample was collected. Although HZ is usually diagnosed clinically, the unusual extent of the lesions and the need for hospitalization make the absence of PCR confirmation a limitation of this report.

### 2.3. Therapeutic Intervention and Follow-Up

**Figure 2:**
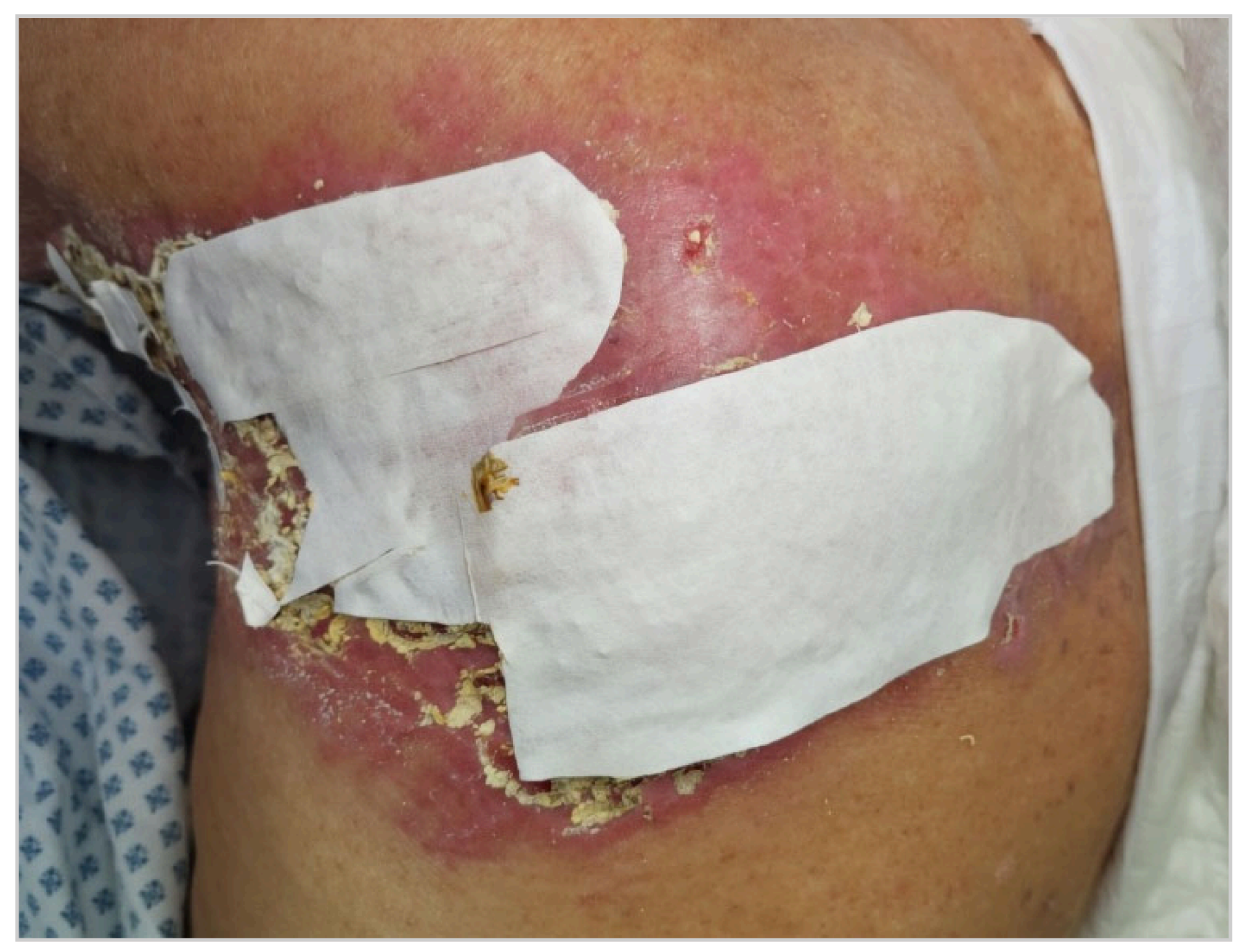
Polylactic acid membrane placed directly over the wound covering the whole lesion.

The patient was admitted for inpatient management. A wound culture was obtained to rule out secondary bacterial infection before any dressing was applied; results were negative for pathogenic growth. Oral valacyclovir (1000 mg every 8 h) was continued to arrest ongoing viral replication. Analgesia was escalated to include systemic opioids for the nociceptive component of his pain (morphine 2.5 mg intravenously every 4 h as needed, then converted to oral tramadol 50 mg every 8 h at discharge) and oral pregabalin (started at 75 mg every 12 h; the dose was tapered from the second follow-up visit) for acute neuropathic pain and hyperalgesia. For wound care, the wound bed was gently cleansed with sterile saline to remove debris, exudate, and residual topical ointment from prior outpatient treatment, avoiding mechanical trauma to the friable surrounding skin. A single ST250 membrane was then placed directly over the entire denuded area (Figure 2). The membrane adhered spontaneously to the moist wound bed without requiring additional adhesive tape or bandaging. A secondary layer of Vaseline-impregnated gauze was placed over the membrane to minimize mechanical friction against clothing and bedding, and the patient tolerated the application without any acute increase in pain.

**Figure 3:**
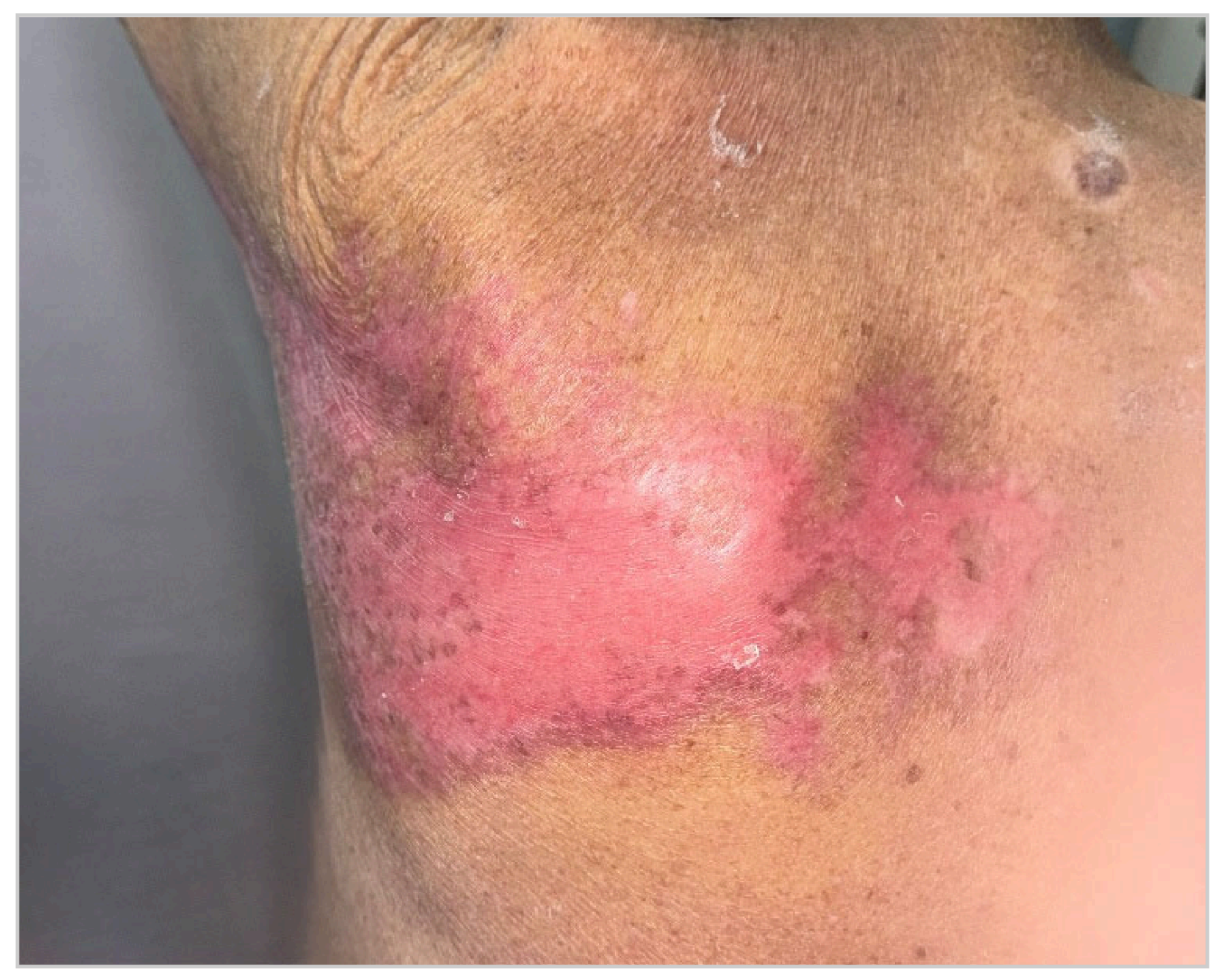
Complete re-epithelialization at the 12-day follow-up visit.

He was discharged after 24 h of inpatient observation, continuing oral valacyclovir and his analgesic regimen. At outpatient follow-up seven days after membrane placement, his pain had decreased to 2/10 on the NRS (from 9/10 at admission) while he continued the same analgesic regimen, and the membrane remained adherent with visible early re-epithelialization at its margins. At the second follow-up visit, 12 days after application, the membrane had fully resorbed and the wound demonstrated complete re-epithelialization (Figure 3) without dehiscence, exudate, or clinical signs of infection; analgesic tapering was initiated at this visit. By the third week, his caregivers reported that he had resumed dressing and bathing independently and was sleeping without positional discomfort related to the healed area. At a final follow-up approximately six weeks after presentation, the healed skin showed no hypertrophic scarring, keloid formation, or persistent dyspigmentation and the patient reported no residual pain at the treated site; because PHN is generally defined as pain persisting for at least three months after rash onset, this six-week assessment can neither exclude PHN nor demonstrate its prevention. No adverse reactions to the membrane were observed at any point during follow-up. In this report, complete re-epithelialization was defined as complete epidermal coverage of the wound surface, without residual erosion, exudate, or visible membrane, as assessed clinically at each visit by the treating wound-care physician (M.A.M.-J.) The clinical timeline is summarized in Table 1.

### 2.4. Patient Perspective

The patient described the onset as a burning sensation that rapidly became severe pain, to the point that “even the touch of my clothing was unbearable,” leaving him unable to dress or bathe. After the membrane was applied, he reported immediate relief, attributing it to protection of the wound from friction, and over the following days his pain decreased, allowing him to sleep and eat normally. He noted that the membrane was absorbed on its own and that the skin healed without significant scarring. He expressed gratitude to the medical staff and reported full recovery.

## 3. Discussion

### 3.1. Clinical Course After Application of a Polylactic Acid Membrane to a Herpes Zoster Lesion

In this patient, a single application of a polylactic acid membrane was followed by a reduction in pain from 9/10 to 2/10 on the NRS within seven days and complete wound closure by day 12, without the need for repeated dressing changes. These observations are descriptive. The patient simultaneously received antiviral therapy and systemic analgesics, and the natural course of HZ, in which pain and epithelialization improve spontaneously over weeks, is an important alternative explanation for both outcomes; the temporal association with the membrane therefore does not show that the membrane was responsible.

A published case describing hydrocolloid dressing use in a patient with severe HZ-related pain and extensive epidermal damage reported a reduction from 9/10 to 4/10 on the Visual Analogue Scale (VAS); because the pain scale (VAS versus NRS), the patient, wound extent, and concomitant treatments differ, the two pain trajectories cannot be directly compared [16]. In the broader literature review by Bolton and colleagues summarizing patient-centered HZ wound outcomes across published case series, healing time with moisture-retentive dressings ranged from 2 to 7 weeks, compared with 9 to 10 weeks when lesions were managed with simple air exposure or dry gauze [8,11]. Our patient’s 12-day time to complete closure is shorter than the range reported in that review; this descriptive comparison should not be read as evidence of superiority, because populations, lesion depth, and definitions of healing may differ across reports and the natural course of HZ is a competing explanation.

These comparisons should be interpreted cautiously, as they involve different dressing technologies, wound sizes, and patient populations, and none derive from a head-to-head controlled trial. However, the available observations, namely better outcomes reported with occlusive, moisture-retentive dressings than with dry or air-exposed management, and the course seen in our patient, are compatible with, but do not test, the underlying rationale that HZ erosions behave as partial-thickness wounds that benefit from the same principles of moist wound healing established for burns and other cutaneous defects [11,16].

### 3.2. Pain Control

The pain course observed in this case is compatible with mechanisms described for ST250 in burn wounds, where it has been shown to significantly reduce pain scores compared with paraffin gauze and other comparator dressings in randomized clinical trials [12,13]. ST250 is a CE-certified, semi-permeable, absorbable alloplastic skin substitute that is highly permeable to oxygen, limits evaporative water loss, and controls the accumulation of inflammatory exudate. Because it adheres to and effectively becomes part of the wound bed rather than requiring removal and reapplication, it avoids the repeated mechanical trauma to exposed nerve endings that occurs each time a conventional gauze dressing is peeled away from denuded dermis, a mechanism that could have contributed to the absence of further analgesic escalation and to the tapering of opioid and pregabalin from the second follow-up visit; however, the contribution of the membrane cannot be separated from that of the concurrent antiviral and systemic analgesic therapy or from the natural resolution of HZ pain [12]. Lactate is recognized as a regulator of angiogenesis and inflammation in wound healing, and it has been hypothesized that lactate released as polylactic acid-based dressings degrade could contribute to healing. However, the amount of lactate actually released into the wound bed by PLA-based matrices, and its effect on healing in vivo, have not been established, and no lactate measurement was made in this patient; this mechanism therefore remains speculative [15].

Direct comparators for skin-substitute use in HZ specifically are scarce, which itself reflects the evidence gap this case seeks to address. The most relevant prior report, discussed above, is the hydrocolloid dressing case published by Kiedrowski, describing an elderly woman with recurrent HZ and extensive epidermal damage whose VAS pain score fell from 9/10 to 4/10 with hydrocolloid occlusion, alongside improved quality of life and accelerated healing relative to her own prior HZ episodes managed conventionally [16]. Differences in pain scales, wound characteristics, concomitant analgesia, and follow-up preclude any inference that the membrane offers an advantage over hydrocolloid dressings. A single-application course, which avoids repeated dressing changes, is a practical feature that merits evaluation in controlled studies.

Beyond HZ, ST250 and related polylactic-acid-based matrices have an established comparative track record against other wound-care modalities. Compared with collagen dressings and acellular xenografts in chronic wounds such as diabetic foot ulcers and venous leg ulcers, polylactic acid-based composite dressings have been reported to shorten healing time and improve healing rates [17]. In burns specifically, a randomized bicentric trial found that ST250 produced significantly lower pain scores than the comparator dressing with no significant difference in healing time or re-epithelialization [13]. A 15-year pediatric cohort further reported favorable adherence to the wound bed and a reduced need for frequent dressing changes across nearly 2100 patients treated with ST250 for second-degree burns [18]. Taken together with the 2025 European case series describing successful use of a polylactic acid matrix in chronic, non-burn wounds [15], this body of evidence provides a rationale for evaluating the extension of polylactic-acid-based membranes to an acute, painful dermatomal wound such as HZ, even though the present report constitutes, to our knowledge, the first such application described in the literature.

### 3.3. Limitations and Clinical Practice Implications

This report describes a single patient, and as with any case report, the observed outcome cannot establish efficacy or be generalized without controlled comparison. Pain scores were recorded by clinical staff at scheduled visits rather than through a structured, blinded pain diary. Pain and wound healing were assessed while antiviral and systemic analgesic therapy were being given, and the natural course of HZ is an alternative explanation for both the pain reduction and the re-epithelialization, so no causal effect of the membrane can be inferred. The diagnosis was clinical, without VZV PCR confirmation; follow-up lasted about six weeks and therefore cannot address post-herpetic neuralgia; and no outcome measure other than the NRS was used. Regarding regulatory status, ST250 is CE-marked and marketed in Germany and across the European Union for burn care. Standard Suprathel, the same product in a thinner version (100 μm thickness), is also CE-marked and holds U.S. FDA 510(k) clearance (K090160). According to the manufacturer, standard Suprathel is also registered for burn and wound care in other countries, including Brazil and Colombia. ST250 is currently registered for use in Europe only; in the rest of the countries it is still considered an investigational product, and its registration is in process. Where ST250 is already authorized for burns, its use on a superficial partial-thickness HZ wound extends an established indication rather than constituting an experimental protocol. Deep wounds after severe HZ may be too infrequent for the manufacturer to seek a specific indication, and case reports such as this one can help alert clinicians to this application. Nonetheless, the magnitude and consistency of the clinical response (rapid pain reduction, single-application wound closure, and an uneventful healing course in a patient of advanced age with an unusually extensive lesion) are hypothesis-generating and support further investigation, but do not establish efficacy. Vaccination remains the most effective strategy for preventing HZ and its complications, and its promotion should remain a public health priority, particularly given the rising incidence associated with global population aging [3,4]. However, patients who are unvaccinated, who decline vaccination, or who develop HZ despite vaccination will continue to require an effective acute wound-care strategy. Given the existing evidence base for ST250 in burns and chronic wounds, and the observations from this case alongside the single prior hydrocolloid report, we suggest that polylactic-acid-based membranes warrant evaluation in a prospective, ideally controlled, series of HZ patients particularly those with extensive dermatomal involvement, in whom conventional gauze-based care is often poorly tolerated and in whom the risk of prolonged pain and delayed healing is highest.

## 4. Conclusions

In this patient with extensive thoracic HZ, a single application of a polylactic acid membrane (Suprathel^®^250) was followed by a reduction in pain (from 9/10 to 2/10 on the NRS within seven days) and complete wound closure by day 12, without adverse events or scarring. Because the patient concurrently received antiviral and systemic analgesic therapy and HZ lesions often improve spontaneously, this single uncontrolled observation does not establish efficacy or comparative benefit over other dressings. It supports further investigation, ideally in prospective controlled studies, of polylactic acid membranes as a wound-care option for extensive HZ erosions.

## Figures and Tables

**Table 1:** Clinical timeline (day 0 = admission and membrane application).

Time	Event
About 7 days before admission	Onset of left thoracic (T6–T8) HZ rash; outpatient treatment with oral acyclovir 500 mg every 4 h while awake, started on day 2 of the rash and lidocaine 5% patches
Day 0 (admission)	Emergency department visit with progressive pain (NRS 9/10), vomiting, and inability to tolerate oral intake; erosions of 12.5 × 24 cm; CRP 40.3 mg/L; wound culture negative; valacyclovir 1000 mg every 8 h continued; systemic opioid and pregabalin added; single ST250 membrane applied
Day 1	Discharge after 24 h of observation on oral valacyclovir and analgesics
Day 7	NRS 2/10; membrane adherent with early marginal re-epithelialization
Day 12	Membrane fully resorbed; complete re-epithelialization (Figure 3); analgesic tapering started
Week 3	Independent dressing and bathing; sleeping without positional discomfort
About week 6	Final follow-up: no hypertrophic scarring, keloid, or dyspigmentation; no residual pain at the treated site; no adverse reactions

## Data Availability

The original contributions presented in this study are included in the article. Further inquiries can be directed to the corresponding author.

## Abbreviations

The following abbreviations are used in this manuscript:

HZ	Herpes zoster
NRS	Numeric Rating Scale
PHN	Post-herpetic neuralgia
ST250	Suprathel^®^250
VAS	Visual Analogue Scale
VZV	Varicella-zoster virus
